# Bimanual or Combined Turning

**Published:** 1877-11

**Authors:** 


Bimanual or Combined Turning.—When at this time I undertake a chapter on operative midwifery, the subject of which has in former and oft-repeated discussions led to apparently satifactory conclusions, it is the observation that certain gained and generally accepted advances, made within the past twenty years, find altogether too limited application in practice.

So far as I have been enabled to observe, it is yet the custom of the majority of obstetricians, as soon as the external manipulations have not led to a satisfactory presentation of the fœtus, to introduce at once the whole hand into the uterus, there to grasp either the feet or the head in order to turn the child. Certainly a large number of cases could be related, the entirely satisfactory result of which, would encourage a repetition of the foregoing procedure under similar circumstances. Yet we must not lose sight of the dangers which are undoubtedly connected in a far greater degree with this operative procedure, than are connected with the manipulations made upon the abdomen, or with their assistance, with a partially introduced hand into the uterus.

Regarding the advantages connected with such a relatively inferior procedure, probably all agree. Furthermore, I fully agree with the principal text-books, so far as they teach the combined inner and outer turning for the presentation of the head; less, however, can I agree with the views as expressed in these text-books, and the more recent operative teachings, regarding the bimanual or combined turning by the feet. This is more or less carefully discussed and recommended, but its applicability is regarded only as an attempt, and entirely confined to those cases in which the waters have not yet broken, and the fetus is moveable within the fluid.

It is claimed that we should not delay with combined turning, in cases where any danger demands immediate delivery, the combined method being too uncertain; and the combined turning should only then be made when the mouth of the uterus is partially dilated, while in complete dilatation the combined turning should not be entertained, or only then when the version of the fœtus can be readily accomplished in this manner. Others give opinions still less positively, recommending the execution of the combined turning where version is indicated at a time when the external manipulations have failed, the inner, namely, with the entire introduced hand, can not yet be executed. When, under these circumstances, the practitioner is already made doubtful whether combined version is only a theoretical teaching, devoid of all practical basis, its acceptance will be much hindered when, as has recently occurred, that such version is condemned by Professors Spiegleberg and Kleinwachter, in just those cases where its advocates specially recommended it, viz: in placenta prævia.

Since the beginning of my clinical experience I have given the method of combined turning my especial attention and study, and have omitted no opportunity to test its applicability. Within the past four years, to the fall of 1876, as assistant of the Berlin Clinic and in private practice, I have made one hundred and eighty-one versions by the feet. The indications of these versions were partially oblique and transverse positions, partly frontal presentations, preventing apparently the advancement of the head, on account of contraction of the pelvis, or a prolapsed funis, or placenta prævia, and other reasons demanding the conclusion of the labor.

In the majority of the cases I was obliged to operate a considerable time after the rupture of the waters, as the nature of an out-door clinic brings with itself. In all these cases external manipulations were without any influence upon the position of the fœtus; and in all I undertook the combined turning, and concluded one hundred and twenty out of the one hundred and eighty-one cases in this manner.

By combined turning by the feet, I understand that procedure by which the greater part of the hand is introduced into the vagina only; then introducing one or two fingers, or in the most difficult cases four fingers, into the os uteri, but always excluding the thumb, and pushing the presenting part to one side of the internal os uteri, at the same time energetically supporting by the externally applied hand, seize or pull down a foot or a knee, which is to be pushed down by the externally applied hand; while by aid of the drawn-down foot the breech is guided into the pelvic brim, the externally applied hand completes the changing of the child by pushing the head to the fundus.

To accomplish this version, the female, with few exceptions, was laid on that side upon which the fœtal feet lay, introducing from behind the hand *heteroime* to that side. As a rule, even when the mouth of the womb was entirely dilated, only two fingers were introduced into the cervix and passed beyond the os uteri. The firmer the impaction of the presenting part of the fœtus, and the more difficult to push the same up, three fingers, or the half of the hand, was used in the first act of the version, for the dislodgment of the presenting part. The dislodgment of the fœtus by this method was not always easily accomplished; especially at times did it cause serious difficulty to push the feet downward by external manipulations.

Nevertheless, out of the one hundred and twenty cases, I have never seen any injury done to the mother by this version. Three of them died shortly after delivery from lesion of the cervix, the effect of forced extraction in placenta prævia; ten in child-bed, and nine of these from septicæmia after placenta prævia. In how far the child has sustained damage by the combined version, cannot, from the manifold dangers attending extraction, be readily ascertained. Forty-four of the children were still-born; of these seventeen were positively dead before version was undertaken, two were not viable, in two embryotomy had to be performed, and twenty-one died during the delivery.

The only obstacle which the combined version cannot overcome, is the firm contraction of the uterus upon the fœtus. The loss of the amniotic fluid alone cannot be of controlling influence in the decision of these cases; for often I have been enabled, some days after the waters had been discharged, to execute the version of the fœtus by the combined method.

When the contraction is of the nature that it requires the introduction of the whole hand to accomplish version, we are only able to answer the question as to the value of this method after production of narcosis; for how often do we not observe how rapidly the versions succeed by the combined manipulations when, under the influence of chloroform or morphia, we begin to manipulate, while just before the fœtus seemed to lie entirely immovable under contractions. Under the influence of the above named agents the irregular spasmodic contractions cease as a rule, and one or two more fingers can be more readily and delicately introduced along the head or the compressed part than the whole hand. Less easily can the contraction upon the fœtus be overcome by long-continued, regular uterine activity, especially if the presenting part has been considerably pushed down.,

These are the cases in which the combined procedure for version was without result. Here it was that the introduction of the entire hand alone saved me from the necessity of embryotomy, which latter I have until yet not been obliged to perform, notwithstanding the fact that I have met with quite a number of very desperate cases. Only then should we desist from longer continued efforts by the combined method, when, by long continued labor, the womb is firmly contracted upon the fetus.

The other contra-indications named by authors I have not found verified as such. It is further claimed that the combined turning does not give immediate desired results in cases of sudden danger. I have never more quickly and safely executed version than by the combined method. It is claimed that in prolapse of the hand the combined method cannot be executed. In eleven of my cases there was prolapse of the arm, and the same has never bothered me by the combined version. Furthermore it is claimed that the dead fœtus cannot be turned in this manner. In seventeen cases of ascertained death, in which many of the fœtus were entirely rotten, I have employed the combined turning.

Finally, it is claimed that the combined version was not practicable in placenta prævia. In twenty-nine of these cases I had to execute version, and in all I turned the fœtus by combined inner and outer manipulations, irrespective of whether the placenta was centrally or laterally attached. In all cases the operation was connected with an insignificant hemorrhage; and had I been satisfied with the turning of the child, and not allowed myself to be influenced by the suggestions of others, depending upon the dilatability of the mouth of the womb, and had I not proceeded in these cases to immediate extraction, I should not so seriously have injured those three of the above mentioned patients. Especially in placenta prævia I have learned to value the gentleness of the combined version, and can therefore recommend its use in just such cases.

It may be objected that even according to my figures I have found it necessary to employ the entire hand in thirty-three per cent. of the cases, which seems like a desertion of the combined method. But when it is considered that the number of cases in which the combined method was employed would have been increased by about thirty had I not left the easier cases to the practitioners assisting me; and, furthermore, that in nineteen out of sixty-one cases there had occurred most remarkable errors before the task came to me to execute the delivery, and as already before stated at times many hours had elapsed before I could reach the patient—it will be allowed that under more favorable circumstances the number of cases requiring the introduction of the entire hand, as an ultimate refuge, would have been greatly diminished.

I am forced to the conclusion, when reviewing the number of cases of version, that the combined method deserves a far greater application than it has hitherto had. Not only should it be attempted in cases of partially dilated os uteri, or in cases of an easily moved fœtus, nor must we go to work timidly and doubtfully, but employ the same patience and perseverance which we have hitherto employed in versions by introducing the entire hand; then we will be enabled to more surely save our patients from the evil results which the version of the fœtus in utero by the inner manipulations not very infrequently produces.—*American Practitioner.*

